# Exploring the correlation of epidemiological and clinical factors with facial injury severity scores in maxillofacial trauma: a comprehensive analysis

**DOI:** 10.3389/froh.2025.1532133

**Published:** 2025-02-17

**Authors:** Weronika Michalik, Julia Töppich, Adam Łuksza, Jakub Bargiel, Krzysztof Gąsiorowski, Tomasz Marecik, Paweł Szczurowski, Grażyna Wyszyńska-Pawelec, Michał Gontarz

**Affiliations:** 1 Students’ Scientific Group of the Department of Cranio-Maxillofacial Surgery, Jagiellonian University Medical College, Cracow, Poland; 2 Department of Cranio-Maxillofacial Surgery, Jagiellonian University Medical College, Cracow, Poland

**Keywords:** Facial Injury Severity Scale, panfacial fractures, maxillofacial trauma management, epidemiology, panfacial fracture (PF)

## Abstract

**Introduction:**

The Facial Injury Severity Scale (FISS) provides a numerical value based on individual fractures that can be a valuable tool for management of maxillofacial trauma patients. The aim of this study was to evaluate the association of epidemiological and clinical factors with facial fracture patterns and their correlations with FISS.

**Methods:**

A retrospective study was conducted based on 511 medical records from a 4-year period of patients with facial trauma who underwent open reduction internal fixation (ORIF) under general anesthesia. Fracture patterns were categorized into 3 anatomic subunits: upper, middle and lower face. Single-unit and panfacial fractures groups were analyzed separately. Data regarding demographics, hospitalization, etiology of injury, fracture site and complications were collected. The overall risk of fracture within the viscerocranium requiring an ORIF was presented in graphical form.

**Results:**

Single-unit fractures were more typical in younger patients. There was a significant association between FISS score and traumatic etiology, hospitalization time, length of surgery in each group (*p* < 0.001). For panfacial fractures, FISS >6 indicated prolonged surgery (>2 h) and hospitalization (>1 week).

**Discussion:**

Despite the questionable clinical utility of FISS, classifying maxillofacial trauma can facilitate comprehensive treatment planning and multidisciplinary collaboration, particularly in complex cases such as panfacial fractures.

## 1 Introduction

Facial fractures are a major challenge in trauma care due to the complex structure of this anatomical area. Correct classification is essential for effective treatment, as these trauma cases are particularly severe and require comprehensive management. The facial skeleton can be divided into three subunits: the upper third, comprising the frontal bone, fronto-orbital bandeau and sphenoid sinus; the middle third, comprising the orbit, zygoma, ethmoid, nose, maxilla and maxillary alveolar ridge; and the lower third, comprising the mandible and alveolar ridge. The middle third is subdivided in some classifications into the upper midface, which includes the lateral and medial orbital wall, orbital floor, naso-orbito-ethmoid area, zygomatic arch and nasal bone; and the lower midface, which includes the maxillary sinus, bony palate and maxillary alveolar ridge (1, 2). In 1989, Markowitz provided a unique definition of combining all facial subunits to diagnose a panfacial fracture (PF) (3). However, the most commonly used definition of PFs is that they involve injuries to two of the three (or three of the four in some interpretations) subunits of the facial skeleton.

Accurate classification of facial fractures is critical for individualizing treatment and improving patient outcomes. The Facial Injury Severity Scale (FISS) is a valuable tool for assessing facial injury severity and planning surgery, particularly in emergencies (4). Higher FISS scores are predictive of greater injury severity, and studies by Bagheri et al. have shown significant correlations between FISS scores and treatment costs (4). Some studies have shown that the FISS scale is a valuable predictor of prolonged hospital stay, the need for surgery and the involvement of multidisciplinary care (5, 6). Different factors influence the characteristics of PFs, which vary between populations. Studies show significant variations in facial fracture patterns and treatment needs between different age groups and genders. For example, older patients often sustain fractures from falls, while younger people are more likely to sustain fractures from car accidents and violence (7). Socio-economic factors also play a role. People from lower socioeconomic backgrounds are more likely to suffer severe facial injuries due to higher rates of violence and limited access to healthcare (8). Alcohol consumption increases the risk of facial trauma by impairing judgement and coordination (9, 10). The correlation between fracture etiology and FISS score is not so obvious. Lin et al. found no significant difference between the cause of fracture and FISS score in PFs (11). On the other hand, Yamamoto et al. revealed that the highest FISS score in pedestrians injured in motor vehicle accidents was caused by trains, followed by cars and motorcycles (12). The management of PFs often requires multidisciplinary and multistage surgical procedures due to the severity of the patient's post-traumatic state. Therefore, complications such as malocclusion, infection or incomplete healing are more likely to occur postoperatively (13). Lin et al's research shows that patients with higher FISS scores often require multiple surgeries, emphasizing the need for thorough initial treatment and careful follow-up (11).

The aim of this study was to verify the significance of the correlation between FISS scores and epidemiological factors, time of surgery and hospital stay, as well as concomitant injuries and complications associated with different patterns of facial fractures through a retrospective study. In addition, the clinical and therapeutic aspect was compared between the group of patients treated for PFs and single unit fractures (SUFs) of the facial bone.

## 2 Materials and methods

### 2.1 Study design and setting

The medical records of 872 patients with facial injuries who were admitted to the University Hospital of Cracow between January 2020 and January 2024 were retrospectively analyzed. Patients with facial skeletal fractures who underwent open reduction and internal fixation (ORIF) surgery under general anesthesia by a maxillofacial surgeon were included in the statistical analysis.

Exclusion criteria were

- Irrelevant/incomplete medical records
- No consent for hospitalization/surgery
- Death before ORIF
- Surgery cancelled due to patient's general condition
- Patients undergoing ORIF under local anesthesia
- Conservative management of facial fractures

**Figure 1:**
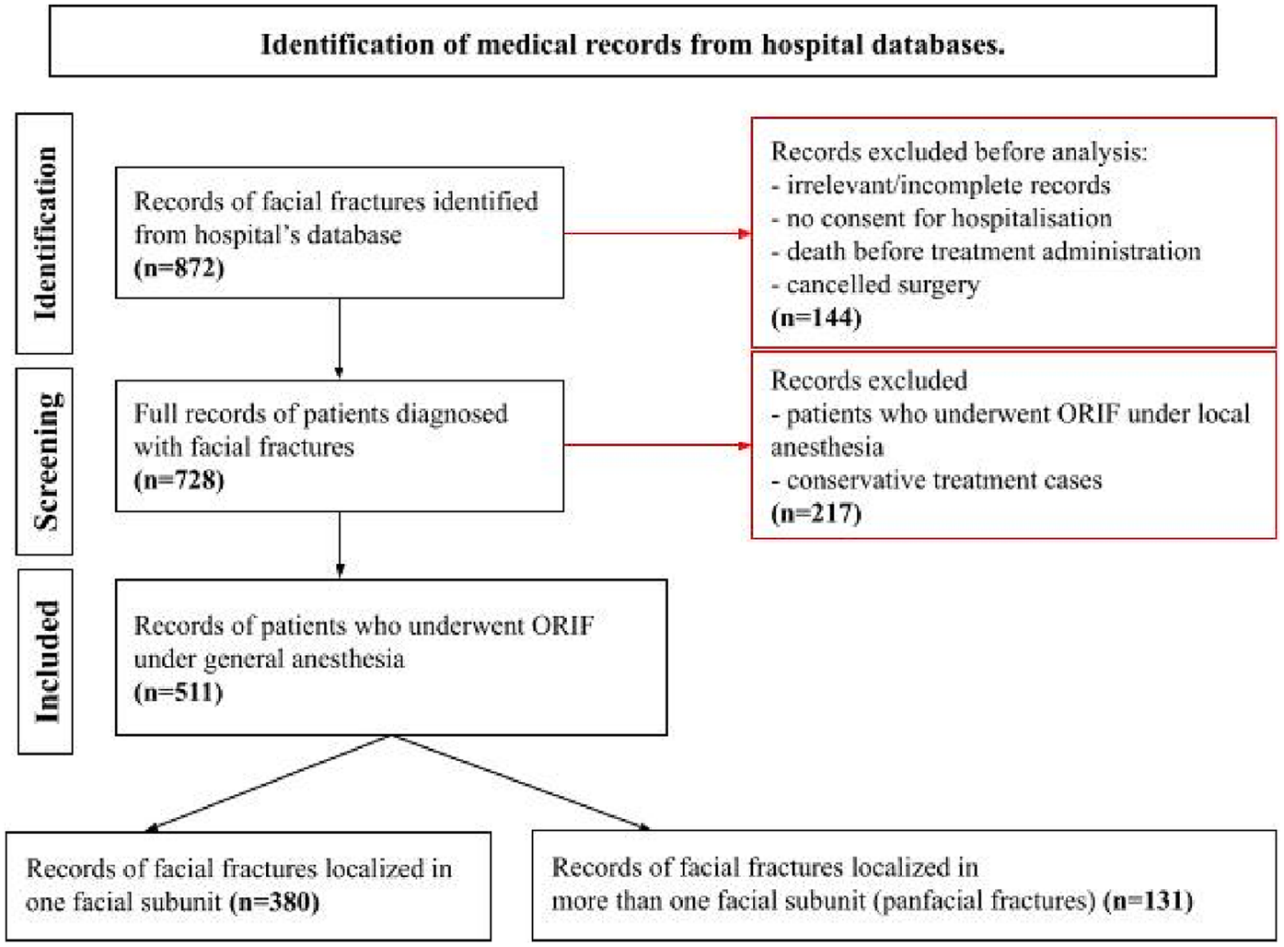
Flow chart describing the process of patients’ selection. Identification of medical records from hospital databases.

The study cohort consists of 511 patients who have been divided into two groups: one group of patients with PFs and the other group of patients with SUFs. The inclusion criteria and exclusion process are characterized in Figure 1. Patient data privacy and confidentiality were strictly maintained throughout the study in accordance with ethical guidelines and regulations.

### 2.2 Investigative factors

**Figure 2:**
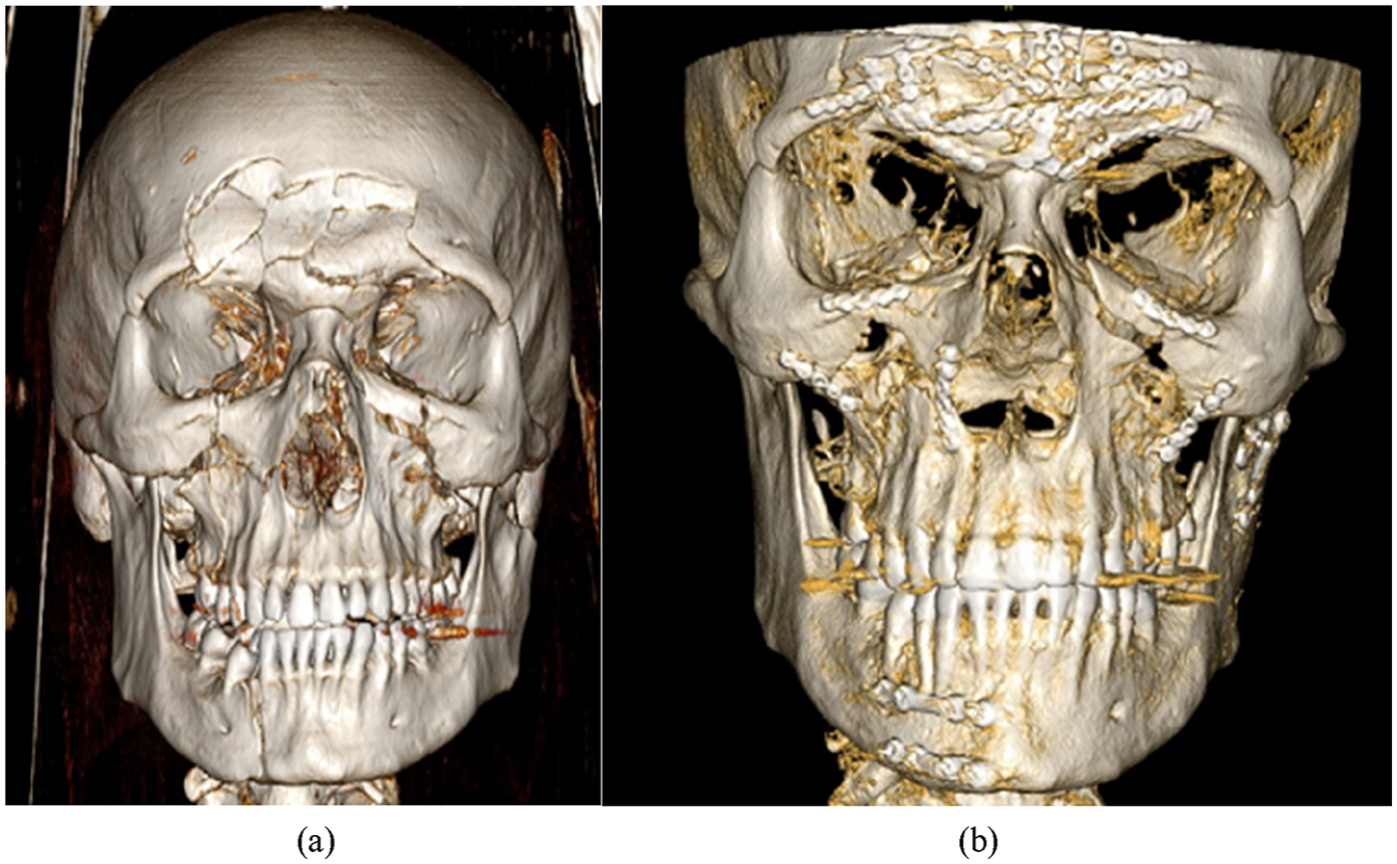
Post-injury **(a)** and post-operative **(b)** 3D reconstructions of CT scans of a 26-year old male patient with UML (upper, middle and lower face) panfacial fractures. The individual calculated FISS score of 12 based on fractures located in: mandibular body (2 points), condyle of the mandible (1 point), bilateral ZMC (2 points), bilateral orbital rim (2 points), displaced frontal sinus (5 points).

All patients were reclassified retrospectively according to the FISS based on initial preoperative diagnosis (Figure 2) (4). A comparison was made between the groups of PFs and SUFs patients with regard to FISS and other factors studied, including:

- Length of hospital stay
- Length of surgery
- Etiology of injury
- Fracture classification
- Postoperative complications
- Number of reoperations
- Concomitant injuries: other skull fractures; cranial nerve injuries; ophthalmological pathologies; central nervous system injuries; limb, spinal and visceral injuries.

### 2.3 Statistical analysis

Spearman's correlation was used to assess the statistical differences between established mechanisms of injury. Pearson's Chi-squared test was used for testing statistical correlation between risk of fracture within particular sites in three established facial subunits. Kruskal-Wallis test enabled assessing statistical correlation between FISS scores and length of surgery and Wilcoxon test was used for testing the significance of FISS scores in different hospitalization times. Data were analyzed using R-Studio 9.3 Build 191.259. A *p*-value of less than 0.05 was considered statistically significant.

## 3 Results

### 3.1 Site of facial fractures

**Figure 3:**
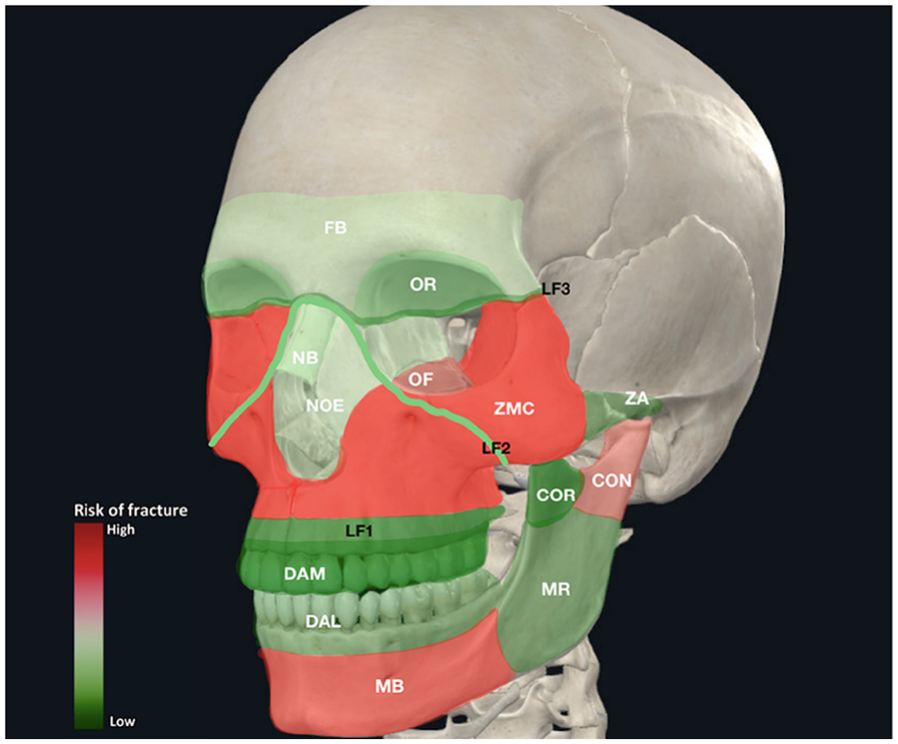
The cumulative heat map illustrates the risk of fracture within established facial subunits in 511 patients (SUFs and PFs), who underwent ORIF under general anesthesia.

The cumulative risk of fracture for all patients in the cohort within each established facial subunit, based on the cumulative number of diagnosed fractures at each site of the facial skeleton, is shown in Figure 3.

**Figure 4:**
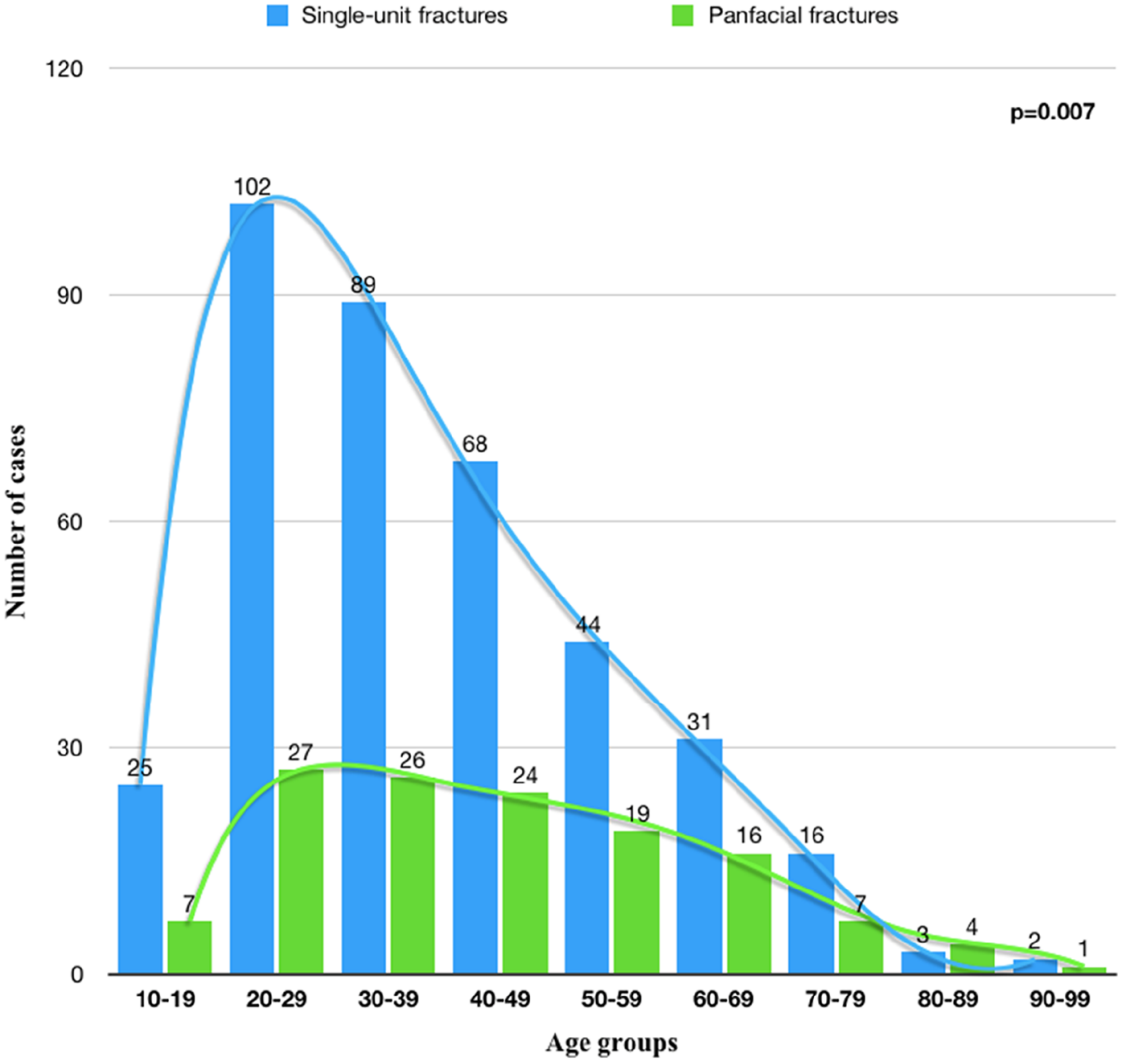
The distribution of SUFs and PFs patients by age group.

In the lower face unit, the mandibular body/angle fractures were the most common (53.6%), followed by fractures of the mandibular condyle (31.8%). The condyle and coronoid process with ramus fractures were relatively more frequent observed in PFs. Conversely, the SUF group showed a higher incidence of fractures in the mandibular body, angle and dentoalveolar processes compared to the PF cases (*p* < 0.0001) Table 1.

**Table 1:** Fracture site depending on facial unit and extent of injury.

Facial subunit	Fracture site		Number of cases			*p*-value
			Total (%)	Single-unit (%)	Panfacial (%)	
**Lower face**	**Total**		**336** (**100%)**	**215** (**100%)**	**121** (**100%)**	<0.0001
	Dento-alveolar	DAL	**27** (**8%)**	24 (11.1%)	3 (2.4%)	
	Body/angle	MB	**180** (**53.6%)**	121 (56.7%)	59 (48.7%)	
	Ramus	MR	**18** (**5.4%)**	9 (4.1%)	9 (7.4%)	
	Condylar process	CON	**107** (**31.8%)**	60 (27.9%)	47 (38.8%)	
	Coronoid process	COR	**4** (**1.2%)**	1 (0.4%)	3 (2.4%)	
**Middle face**	**Total**		**579** (**100%)**	**404** (**100%)**	**175** (**100%)**	<0.0001
	Dento-alveolar	DAM	**7** (**1.2%)**	1 (0.2%)	6 (3.4%)	
	Nasal bone	NB	**57** (**9.8%)**	34 (8.4%)	23 (13.1%)	
	Naso-Orbital Ethmoid	NOE	**52** (**8.9%)**	27 (6.6%)	25 (14.3%)	
	Zygomaticomaxillary complex	ZMC	**216** (**37.3%)**	155 (38.4%)	61 (34.9%)	
	Orbital floor/medial wall	OF	**163** (**28.2%)**	140 (34.7%)	23 (13.1%)	
	Zygomatic arch	ZA	**28** (**4.8%)**	19 (4.7%)	9 (5.1%)	
	Le Fort I	LF1	**21** (**3.6%)**	10 (2.5%)	11 (6.3%)	
	Le Fort II	LF2	**33** (**5.7%)**	18 (4.5%)	15 (8.6%)	
	Le Fort III	LF3	**2** (**0.4%)**	0 (0%)	2 (1.2%)	
**Upper face**	**Total**		**98** (**100%)**	**30** (**100%)**	**68** (**100%)**	0.4190
	Orbital roof/rim	OR	**45** (**45.9%)**	13 (43.3%)	32 (47.1%)	
	Frontal bone/sinus	FB	**53** (**54.1%)**	17 (56.7%)	36 (52.9%)	

Bold text is used to clearly highlight specific sections.

In our cohort, fractures of the midface unit were the most common. The zygomatic-maxillary complex (ZMC) was the most frequently fractured site (37.3%), followed by orbital floor/medial wall fractures (28.2%). Notably, ZMC fractures were also the most common fractures requiring ORIF, followed by fractures of the mandibular body/angle. Additionally, 71.8% of ZMC fractures and 85.9% of orbital floor/medial wall fractures were classified as SUFs. In addition to other midface areas, only the dento-alveolar ridge and LeFort fractures were relatively more common in cases of panfacial fractures (*p* < 0.0001).

In the upper face unit, fractures of the frontal bone/sinus (54.1%) and orbital roof/rim (45.9%) were similarly common and more often observed in panfacial injuries. However, the difference was not found to be statistically significant (*p* = 0.419).

### 3.2 Age distribution

The age of the patients ranged from 12 to 96 years old. The average age was 42.6 ± 18.7 years old. However, the age distribution in the PFs group was more homogeneous than in the SUFs group, with a higher frequency of fractures observed in patients in the second to fourth decades of life (*p* = 0.007) (Figures 3 and 4). A similar frequency of ORIF was observed in elderly patients (>80 years old) with SUFs and PFs.

### 3.3 FISS and fractures characteristics

The etiology of PFs varied by specific fracture configurations are shown in Table 2. Road accidents and falls accounted for 64.1% of traumatic etiologies of PFs, most commonly causing UM and ML fracture patterns. The highest average FISS score was generated by mechanical force (6.88 ± 3.18). The FISS score above 6 indicated prolonged hospitalization time in over 63% of PFs (*p* = 0.012) as well as longer surgical procedures (*p* < 0.0001).

**Table 2:** Average FISS score in relation to traumatic etiology, hospitalization time and length of surgery in single-unit and panfacial fractures.

Variable	Location of facial fractures					
	Single-unit fractures			Panfacial fractures		
	Number of cases (%)	FISS score (±SD)	*p*-value	Number of cases (%)	FISS score (±SD)	*p*-value
Mechanism of injury			<0.0001			<0.0001
Road accident	**75** (**19.7%)**	1.93 (±1.52)		**41** (**31.3%)**	5.32 (±2.40)	
Fall	**113** (**29.7%)**	2.22 (±1.55)		**43** (**32.8%)**	4.14 (±2.49)	
Interpersonal violence	**97** (**25.5%)**	2.29 (±1.54)		**22** (**16.8%)**	4.8 (±2.22)	
Sport	**14** (**3.7%)**	1.29 (±0.79)		**1** (**0.7%)**	5 (±0)	
Mechanical force	**36** (**9.5%)**	2.67 (±1.78)		**8** (**6.1%)**	6.88 (±3.18)	
Other	**45** (**11.9%)**	2.33 (±1.53)		**16** (**12.2%)**	4.47 (±2.33)	
Hospitalization time in days			0.001			0.012
≤6	**244** (**64.2%)**	1.99 (±1.41)		**48** (**36.6%)**	4.54 (±2.63)	
>6	**136** (**35.8%)**	2.58 (±1.74)		**83** (**63.4%)**	6.04 (±2.48)	
Length of surgery in minutes			<0.0001			<0.0001
≤60	**196** (**51.6%)**	1.7 (±1.21)		**44** (**33.6%)**	3.79 (±2.15)	
(60–120)	**143** (**37.6%)**	2.51 (±1.55)		**34** (**25.9%)**	4 (±1.15)	
≥120	**41** (**10.8%)**	3.28 (±1.98)		**53** (**40.5%)**	6.26 (±2.39)	

Bold text is used to clearly highlight specific sections. FISS, facial injury severity scale; SD, standard deviation.

On the other hand in SUFs falls and interpersonal violence accounted for 55.2% of traumatic etiologies in this group, most commonly affecting the middle face region. The highest FISS values for the upper face were caused by interpersonal violence followed by mechanical force. The FISS score above 3 correlated with longer hospital stay (*p* = 0.001) and duration of surgery (*p* < 0.0001).

### 3.4 Single-unit vs. panfacial fractures

Table 3 presents a cumulative comparison between SUFs and PFs cases. Among all patients, 419 (82%) were male, 92 (18%) were female, resulting in a male to female ratio of 4.6:1. Men more often presented higher FISS values than women (*p* = 0.025). Supposedly, the FISS score reached higher values in the PFs with the maximum value of 13 (*p* = 0.0003).

**Table 3:** Comparison of single-unit and panfacial fractures.

Variable	Number of cases, total (%)	Single-unit fractures, *n* (%)	Panfacial fractures, *n* (%)	*p*-value
**Sex**				**0.025**
**Total**	**511** (**100%)**	**380** (**100%)**	**131** (**100%)**	
Male	419 (82%)	306 (80.5%)	112 (85.5%)	
Female	92 (18%)	74 (19.5%)	19 (14.5%)	
**FISS score**				0.0003
Range	–	1–7	2–13	
Average value	–	2 ± 1.55	4.87 ± 2.52	
**Mechanism of injury**	**511** (**100%)**	**380** (**100%)**	**131** (**100%)**	<0.0001
Road accident	116 (22.7%)	75 (19.7%)	41 (31.3%)	
Fall	156 (30.5%)	113 (29.7%)	43 (32.8%)	
Interpersonal violence	119 (23.3%)	97 (25.5%)	22 (16.8%)	
Sport	15 (2.9%)	14 (3.7%)	1 (0.7%)	
Mechanical force	44 (8.6%)	36 (9.5%)	8 (6.1%)	
Other	61 (12%)	45 (11.8%)	16 (12.2%)	
**Concomitant injuries**	**511** (**100%)**	**380** (**100%)**	**131** (**100%)**	0.0008
Other skull fractures	12 (2.3%)	7 (1.8%)	5 (3.8%)	0.0005
Cranial nerves	15 (2.9%)	10 (2.6%)	5 (3.8%)	0.005
Ophthalmologic	46 (9%)	36 (9.5%)	10 (7.6%)	0.1280
Skin laceration	113 (22.1%)	80 (21.1%)	33 (25.2%)	0.0055
Central nervous system	45 (8.8%)	26 (6.8%)	19 (14.5%)	0.002
Spine injuries	28 (5.5%)	18 (4.7%)	10 (7.6%)	0.001
Limbs	46 (9%)	27 (7.1%)	19 (14.5%)	0.0003
Visceral organs	28 (5.5%)	13 (3.4%)	15 (11.4%)	0.00001
**Early surgical airway management**	37 (7.2%)	4 (1%)	33 (25.2%)	<0.000001
**Postoperative complications**	41 (8%)	26 (6.8%)	15 (11.4%)	0.0037
**Reoperations**				0.892
1	27 (5.3%)	20 (5.2%)	7 (5.3%)	
>1	7 (1.4%)	5 (1.5%)	2 (1.5%)	

Bold text is used to clearly highlight specific sections.

In terms of the mechanisms of injury: road accidents, falls and other unspecified causes resulted more often than ¼ in PFs, whereas interpersonal violence, mechanical force hits and sports-related injuries were associated in over 80% with SUFs.

Among all patients, 333 major concomitant injuries were observed in 228 patients (44.6%). The most common was skin laceration, accounting for 22.1% of all cases, followed by ophthalmologic pathologies (concussion of the retina, hyphema, lens dislocation or eyeball rupture) (9%), limb injuries (9%), central nervous system (CNS) pathologies (cerebral contusion, epidural and subdural hematoma, subarachnoid hemorrhages) (8.8%). Other skull fractures (mainly affecting the skull base), CNS pathologies, injuries of the limbs and visceral organs were more often associated with PFs than other concomitant injuries as a result of a severe polytrauma with accompanying panfacial injuries in these cases. Early surgical airway management was required more often in PFs with statistically higher FISS scores (*p* < 0.000001).

Postoperative complications were associated with 6.8% of SUFs and 11.4% of PFs thus the positive correlation with the severity of trauma was noted (*p* = 0.037). No such correlation was found in terms of the need or number of reoperations as in both groups there was a similar tendency for additional surgical intervention.

## 4 Discussion

Classification and management of facial fractures is essential in the management of maxillofacial trauma due to the complex structure of the facial skeleton. Many classification systems have been proposed to effectively guide treatment strategies. Markowitz et al. proposed a classification based on three anatomical landmarks: upper, middle and lower subunits. Although this classification assumes that PFs occur when all three subunits are injured simultaneously, it is not widely used today due to its limitations (3). Yun et al. proposed an alternative classification in which PFs involve at least two of the three subunits—upper, middle and lower face—simultaneously (14). This classification allows for a more comprehensive and standardized approach to the diagnosis and management of severe facial injuries, thereby improving patient outcomes (14). It is also consistent with the Facial Injury Severity Scale (FISS) by Bagheri et al., which was chosen as the objectifying tool in this study due to its effectiveness and simplicity in assessing the severity of facial injuries (4). The FISS has been shown to be highly reliable, particularly in emergency settings where quick assessment is critical (15). As noted by Follmar et al., the complexity of PFs necessitates a reliable classification system, and an understanding of the anatomy and potential injury patterns is essential for effective management (16). The FISS facilitates multidisciplinary collaboration and has been validated in various clinical scenarios (4).

The overall risk of facial fractures was assessed in this study, with SUFs being more common than PFs (Figure 1). This pattern is consistent with existing literature indicating a higher incidence of single unit injuries (17, 18). The midface was the most commonly affected region in both SUFs and PFs, consistent with previous studies highlighting the high frequency of fractures in this area (12, 17).

In the lower face, mandibular body or angle fractures were the most common consisting 53.6% of all fractures within this unit, followed by 31.8% of mandibular condyle fractures. In SUFs, fractures of the body or angle and the condylar process account for 56.7% and 27.9% of lower face unit fractures, respectively. Conversely, condylar process fractures were relatively more common in PFs involving the lower face unit, accounting for 38.8%. Fractures concerning the condyle often coexist with the mandibular body or angle fractures, due to the mechanism of injury. Most studies lead to similar conclusions (18–21), although some differences may result from the fact that in this study fractures to the mandibular symphysis and parasymphysis were counted as a part of the category: “fractures to the body/angle”. Apart from the mechanism of injury and specific patients' predispositions, anatomical features—such as the highest mobility of all facial bones—and muscle attachments contribute to the higher incidence of fractures in these regions (22). Also, the presence of a third molar can allow the force to fully disperse during the occurrence of an angle fracture, creating a point of weakness regardless of whether the tooth is impacted or not (23). In the present study, minor differences in incidence of SUFs and PFs to the lower face were observed.

Fractures to the middle face showed the highest prevalence among all the fractures analyzed in this study. Previous studies lead to similar conclusions concerning the ZMC fractures, although some differences regarding fractures to other midfacial regions can be observed. In the present study, ZMC fractures accounted for 37.3% of all midfacial fractures in both SUFs and PFs. Fractures to the orbital floor/medial wall were the second most commonly observed fractures in the midface unit (28.2%). These observations align with the study by Karikal and Priyank, where the ZMC fractures accounted for 41.8% of all fractures, and orbital floor fractures - 36.6% (24). As the ZMC is situated prominently in the facial skeleton, it is primarily more prone to the fractures, due to a higher risk of exposure to the mechanical forces, compared to other midfacial structures. The incidence of different fracture patterns differs significantly among the studies. In the present study nasal bone fractures were observed only in 9.8% of cases, which stands against the observations in the study by Jin et al., where the nasal bone was fractured the most. These differences may result from the fact that in most cases of isolated nasal bone fractures, conservative treatment or nasal bone reduction under local anesthesia are performed by otolaryngologists (18). In the present study, records of patients who underwent those procedures were excluded, leading to a lower incidence of nasal bone fractures.

In the present study, fractures concerning the upper face were found to be the least prevalent. Furthermore, these fractures exhibited a more than double higher incidence among PFs than SUFs, which is in contrast to the observations concerning the lower and middle face. Consequently, these injuries are typically more severe, particularly in regard to injuries to the central nervous system, skull base and eyeballs. These cases necessitate the involvement of specialists from the fields of neurosurgery and ophthalmology.

The age distribution analysis presented in Figure 3 revealed that the most commonly injured group was 20–29 years old, consistent with existing literature indicating younger individuals' susceptibility to facial fractures due to high-energy injuries, such as interpersonal violence and car accidents (25). A comparable frequency of ORIF was observed in elderly patients (>80 years old) with SUFs and PFs. However, Atisha et al. reported that individuals aged 80 years and older are more prone to SUFs than PFs (26). In elderly patients with comorbidities, treatment often involves no intervention or closed reduction, especially in the absence of functional impairment. This approach is particularly common in mid and upper face fractures, where the primary indication for surgery is aesthetic, and many patients decline surgical procedures (27). Consequently, it is highly probable that fractures in elderly patients were underestimated in our cohort. In contrast, SUFs were more prevalent in younger patients, affecting 78% of this demographic. The physical and mental impairments frequently observed in elderly individuals often result in low-energy accidents, typically falls, which require minimal surgical intervention (25). The distribution of fracture patterns across all age groups generally shows that SUFs correlated stronger with younger age groups in comparison to PFs, which were more equally distributed among all ages, so the trend line is not as steep as in SUFs. The underlying reasons for this phenomenon are explained by the epidemiological differences in mechanisms of injury (Table 2). Interpersonal violence in younger patients led to a substantial amount of SUFs, whereas in older adult patients road accidents resulted in a higher incidence of PFs in this group.

The mechanism of injury affects the treatment strategy and prognosis. High-energy injuries, such as those resulting from road traffic accidents, are frequently associated with complex fracture patterns and significant soft tissue damage, requiring a multidisciplinary approach to ensure optimal outcomes (28). In contrast, low-energy injuries, such as falls, typically result in isolated fractures that can be managed with less extensive surgical intervention or conservative treatment (28). Comprehensive treatment strategies that address both the primary facial fractures and associated injuries are essential. In the present study, falls contributed to the majority of facial fractures, including both SUFs and PFs. However, the pattern of falls was different in the SUF and PF groups. In the SUF group, falls at the same level tend to occur after alcohol consumption. On the other hand, in the PFs group, falls from height were observed, e.g., from scaffolding or during suicide attempts. Interpersonal violence accounted for 25.5% of all SUFs, making it the second most common cause of SUFs. In contrast, road accidents contributed to 31.3% of all PFs cases. Since road accidents are typically associated with high-energy injury mechanisms, they lead to more PFs, as these fractures are primarily caused by high-energy incidents. Conversely, interpersonal violence, generally a lower-energy mechanism, may result in more localized fractures—SUFs. The highest FISS scores were assigned to fractures caused by high-energy accidents, such as those from mechanical force, road accidents, and sports accidents. These higher scores were primarily seen in PFs, which are more severe and thus receive higher FISS scores. Among the SUFs, fractures caused by mechanical force had the highest FISS scores, while interpersonal violence was associated with the second highest. Previous studies have shown some variability in the incidence of specific fracture mechanisms. Earlier research indicates that road accidents are a major contributor to maxillofacial fractures (7, 29, 30).

Brucoli et al. (31) found that assault was the most frequent cause of injury, followed by falls. The discrepancy may be due to the younger patient group in their study. According to the present findings, injuries caused by mechanical force (crushed by a hydraulic press, car or forklift truck) had the highest FISS scores, followed by car accidents and interpersonal violence. Lin et al. (11) showed that mechanical force injuries were assigned on average 11.6 points on the FISS, with the highest scores concerning work accidents of 12.6 points. The mean FISS score for facial fractures caused by mechanical force was 6.75, which was the highest among all categories in the present study. Although the numerical value is lower, this trend is consistent with the observations made by Lin et al. (11) Erdmann et al. (32) reported that car accidents were the most common cause of PFs, while assaults and falls usually resulted in isolated fractures.

Prolonged hospital stays and operation time highlight the resource-intensive nature of treating PFs. In the present study PFs necessitated hospitalization time of more than 6 days and were assigned significantly higher FISS score (*p* = 0.012), whereas in terms of SUFs hospitalization time of 6 days and less was required in 64.2% of all SUFs cases, which scored 1.99 in FISS. Patients with higher FISS scores generally had longer hospitalization periods, highlighting the intensive care required for severe cases (5). Efficient hospital resource management and post-operative care plans are crucial to ensure optimal recovery for these patients.

PFs also necessitated longer operative times—40.5% of PFs required an operative time of 120 min or more. In contrast, over 50% of SUFs required an operative time of 60 min or less. For PFs, a mean FISS score of 6.26 points was assigned, the highest among both PFs and SUFs. However, the assigned score for a particular fracture does not always reflect the complexity of the required treatment. The scoring of lower face fractures was analyzed, revealing that the operation for a mandibular body fracture, despite being valued at 2 points in the FISS, is often much simpler and shorter than surgery for a mandibular condyle fracture, which is assigned only 1 point. The length of the operations is up to one hour for mandibular body fractures and almost two hours for mandibular condyle fractures (especially head of the condyle). Hence, the FISS should not be used as a predictive tool for estimating operative time, despite being statistically significant, as demonstrated in the present study.

Although in both sex groups SUFs dominated, men were more often affected by panfacial fractures than women, and presented generally higher FISS scores (*p* = 0.025). Consistent with previous studies, young men suffered more often from high-energy accidents, while elderly women were injured more frequently by falls (11). According to Ruslin et al., women have a significantly higher risk of facial fractures accompanied by dental injuries than men. The most frequently injured teeth are the maxillary incisors, followed by the mandibular incisors, with traffic accidents identified as the leading cause of dental injuries (33). These findings align with broader epidemiological trends indicating that the causes and severity of facial fractures vary significantly with age, sex, and socio-economic factors. The data suggest that preventative measures should be tailored to specific demographics to reduce the incidence and severity of facial injuries (34).

PFs often require a multidisciplinary approach due to the risk of severe concomitant injuries which may necessitate immediate attention, delaying surgical treatment of facial fractures (12). They also require quick imaging, usually using the CT polytrauma protocols of the whole body. Additionally, CT scanning plays a crucial role in the entire treatment process, including preoperative planning, intraoperative navigation, and postoperative assessment of outcomes (35, 36). The statistical analysis confirmed that patients with PFs, presenting higher FISS scores, had a significantly higher risk of severe concomitant injuries (*p* = 0.0008). This observation was also stated by Lin et al. who indicated that with higher FISS scores, more complex treatments, especially for cervical spine injuries is required (11).

**Figure 5:**
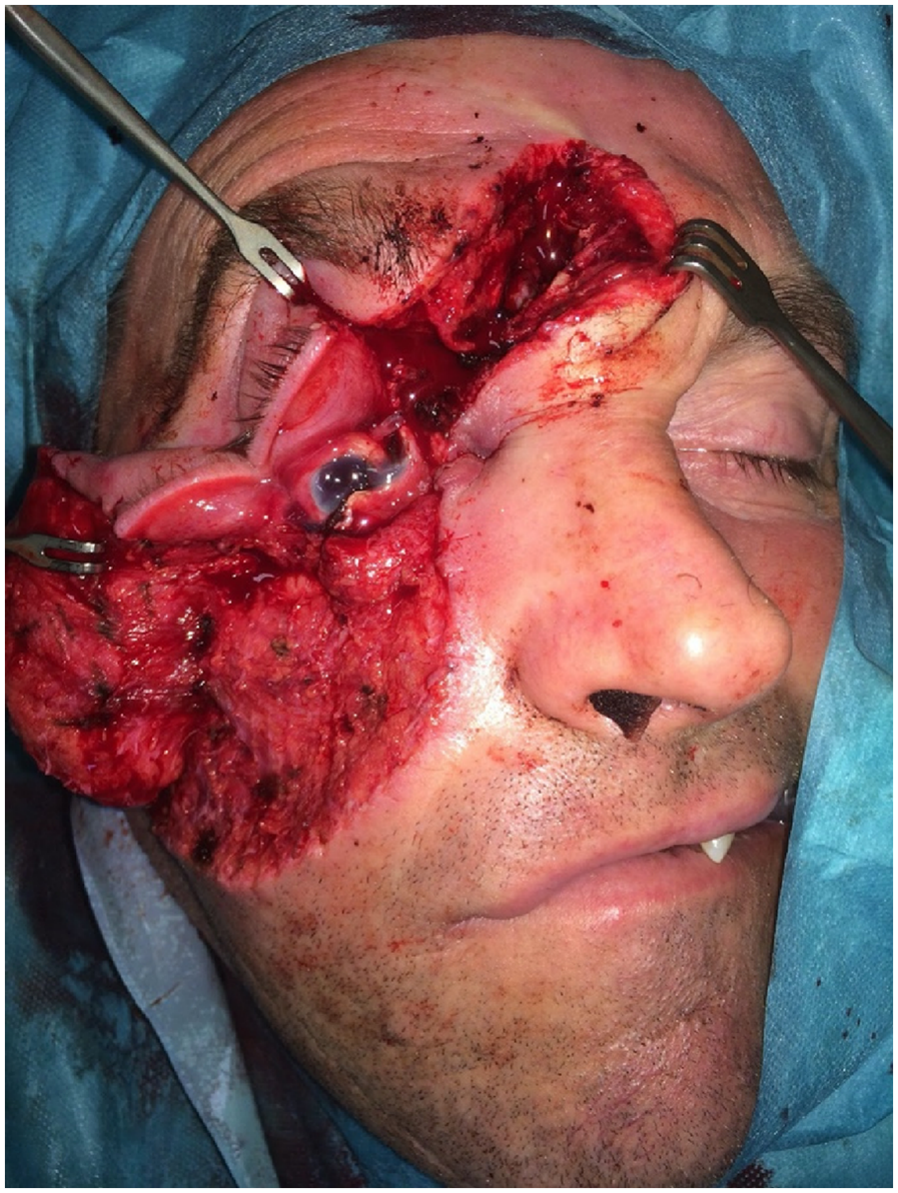
The patient presented with an angle grinder facial laceration with transection of the right eyeball (requiring enucleation), a fracture of the anterior table of the frontal sinus and the upper orbital rim.

Skin lacerations were the most observed injuries, though most cases were minor and required minimal medical intervention. However, injuries involving the eyeballs, skull fractures or central nervous system trauma necessitated neurosurgical and ophthalmological care (Figure 5). Plaisier et al. noted a higher risk of death in patients with PFs, particularly from concomitant neurologic injuries (37). Alvi et al. observed the highest number of cerebral hematomas, predominantly subdural, as coexisting injuries with facial fractures. These patients may require CT angiogram, to assess the risk of blunt cerebrovascular injury, as in case of stroke the risk of death arises from 25 to 50% of cases (38).

Post-injury care with early life-saving steps must be undertaken to ensure that the vital functions are under control. Patients should be stabilized by securing airways and managing bleeding. Cranial nerves, visual acuity and extraocular movements, followed by potential septal hematoma or rhinorrhea should be assessed. Bony step-offs can be found by palpation (39). Although all these steps are necessary to provide effective life-securing treatment, they can be impeded by intubation, cervical collar or lack of patient cooperation. Most patients require oral intubation, because of the upper airway obstruction resulting from foreign body aspiration; tongue fall position; tracheal, laryngeal and facial fractures, as well as regurgitation of stomach contents (40). Depending on the patient's neurological function, or planned surgical treatment, the submental intubation or tracheostomy is implemented. Tracheotomy is preferred in case of patients with lagging neurological function or with multiple surgeries along the way (41). PFs patients required early airway management more often (*p* < 000001) as a consequence of affecting at least one subunit crucial for physiological breathing process, which are the middle or lower face regions.

Postoperative complications almost twice more often concerned PFs patients which correlated with the severity of trauma and the FISS score. In SUFs, dominant ophthalmological complications such as ectropion, entropion, diplopia, enophthalmos, palpebral adhesions, retroorbital haematoma were most typical for single unit midface fractures, considering most of the orbital walls within this unit (42). The second most common was inflammation around the osteosynthesis plates, mainly in lower facial trauma cases. Moreover, there were a few cases of trigeminal and facial nerves dysfunction. Adversely in PFs, bone loss or deformation were among leading postoperative complications followed by inflammation in the close proximity of the surgical field.

Reoperations were often required to address postoperative issues such as inflammation around the osteosynthesis plates, malocclusion, or incomplete bone healing due to osteomyelitis. The procedures that needed to be addressed included revising ORIF, removing osteosynthesis plates, further microvascular reconstruction, or secondary corrections (osteotomies) to improve both function and esthetic result. However, the incidence of reoperations was similar in both SUFs and PFs (around 5% each), so higher FISS scores did not necessarily indicate increased risk of additional surgery. This observation contrasts some studies that proved otherwise (11).

Enhancing the usability and practical implementation of classification scales, such as the FISS, could offer significant benefits. Simplifying these scales would streamline medical procedures, enabling quicker and more accurate assessments, which would ultimately improve patient care and outcomes. There are many examples of scoring systems that can be used as highly informative and reliable tools in daily practice, such as: the Facial Fractures Severity Score (43), the Abbreviated Injury Scale (44), or the Craniofacial Disruption Score (45). However, similarly to the FISS, each scale has its limitations. Recently, a new scoring system—the Comprehensive Facial Injury (CFI) Score—was developed (46). Canzi et al. introduced several improvements in the CFI compared to the original FISS (46). These include a division of the midface that now incorporates the orbital floor and medial wall, as well as a more detailed classification of upper face fractures into frontal sinus/anterior wall fractures, posterior wall/frontonasal duct fractures, and orbital roof/rim fractures. Additionally, soft tissue injuries were included instead of facial lacerations over 10 cm in length. Furthermore, bone atrophy or comminution of fragments adds 3 points to the total score. Overall, the CFI may prove to be a better scoring system for daily use, due to a more precise classification of injuries, as it enables a more precise and objectified evaluation of individual cases. Moreover, it is a highly valuable tool in predicting the mean duration of surgery, length of stay in the surgical ward and in the Intensive Care Unit (46).

## 5 Study limitations

This analysis aims to improve understanding and management of facial fractures; however, it has its limitations. Since, retrospective analysis of the medical records from one hospital facility was performed, the specific treatment options and hospitalization conditions may bias the overall outcome. The limitations, resulting from human error, should be taken into consideration, since both the medical records keeping, and data collection were human-performed. The inclusion criteria, which limit the study to patients undergoing ORIF under general anesthesia, result in the exclusion of a significant proportion of patients receiving other treatments. This may have consequences for the results of the epidemiological and etiological analysis by introducing potential selection bias. Additionally, skin lacerations were not analyzed, even though the FISS assigns 2 points for lacerations of at least 10 cm in length. Since an insufficient number of patients met this criterion, this category was excluded from the analysis. Additionally, due to epidemiological restrictions during the pandemic of COVID-19, only more severe cases were hospitalized. Hence, the incidence of panfacial fractures, in relation to generally less severe single-unit fractures, could be inflated. The etiology of the fractures may also be biased, due to limited interpersonal contacts, contributing to the increase in falls, and the decrease in interpersonal violence. Specific country-related factors may contribute to different observations. However, according to the study by Infante-Cossio et al., describing the impact on the COVID-19 on maxillofacial trauma in Spain, observations concerning the incidence of more severe fractures and the etiology are similar (47).

## 6 Conclusions

In patients with PFs, condyle fractures are a significant concern, occurring around 40% of cases involving lower-face unit. The severity and implications of these injuries are highlighted by the Facial Injury Severity Scale, where a score greater than 6 is associated with a substantial increase in the complexity of treatment. Specifically, such a score predicts the likelihood of prolonged surgical procedures lasting over 2 h and extended hospital stays exceeding 1 week. However, in some cases the allocated points may inadequately underestimate the true severity and clinical impact, particularly in fractures of mandibular condyle. Thus, comprehensive treatment strategies that address both the individual facial fractures and associated injuries are essential.

## Data Availability

The raw data supporting the conclusions of this article will be made available by the authors, without undue reservation.
